# Modulation of DNA-Induced Damage and Repair Capacity in Humans after Dietary Intervention with Lutein-Enriched Fermented Milk

**DOI:** 10.1371/journal.pone.0074135

**Published:** 2013-09-11

**Authors:** Carmen Herrero-Barbudo, Beatriz Soldevilla, Belén Pérez-Sacristán, Inmaculada Blanco-Navarro, Mercedes Herrera, Fernando Granado-Lorencio, Gemma Domínguez

**Affiliations:** 1 Unidad de Vitaminas. Hospital Universitario Puerta de Hierro-Majadahonda, Madrid, Spain; 2 Servicio de Oncología Médica, Hospital Universitario Puerta de Hierro-Majadahonda, Madrid, Spain; 3 Servicio de Bioquímica Clínica, Facultad de Medicina, Universidad Autónoma de Madrid, Madrid, Spain; 4 Departamento de Medicina, Facultad de Medicina, Universidad Autónoma de Madrid, Madrid, Spain; St. Georges University of London, United Kingdom

## Abstract

Dietary factors provide protection against several forms of DNA damage. Additionally, consumer demand for natural products favours the development of bioactive food ingredients with health benefits. Lutein is a promising biologically active component in the food industry. The EFSA Panel on Dietetic Products, Nutrition and Allergies considers that protection from oxidative damage may be a beneficial physiological effect but that a cause and effect relationship has not been established. Thus, our aim was to evaluate the safety and potential functional effect of a lutein-enriched milk product using the Comet Assay in order to analyze the baseline, the induced DNA-damage and the repair capacity in the lymphocytes of 10 healthy donors before and after the intake of the mentioned product. Our data suggest that the regular consumption of lutein-enriched fermented milk results in a significant increase in serum lutein levels and this change is associated with an improvement in the resistance of DNA to damage and the capacity of DNA repair in lymphocytes. Our results also support the lack of a genotoxic effect at the doses supplied as well as the absence of interactions and side effects on other nutritional and biochemicals markers.

## Introduction

Lutein, a plant pigment that is among the best-known carotenoids, is also one of those most widely distributed in frequently consumed fruits and vegetables [1]. Humans are not capable of synthesizing carotenoids *de novo* and their presence in human tissues is therefore entirely of dietary origin. Lutein has no provitamin A activity in humans, although it displays other *in vitro* and *ex vivo* biological activities (i.e antioxidant) that have attracted much attention in relation to human health (i.e. cardiovascular disease, visual function, cancer) [2]–[6].

During past decades, numerous animal and *in vitro* studies have suggested that dietary factors and antioxidants may provide protection against several forms of DNA damage [7], [8]. However, *in vitro* and animal models are not fully appropriate for assessing human *in vivo* bioavailability and function of i.e. carotenoids [9], [10]. Moreover, evaluation of effects on target functions associated with its consumption may be misinterpreted since *in vitro* protocols, with or without cell models, cannot mimic completely the *in vivo* situation, especially regarding the timing, chronic exposure, first-pass metabolism or the effect of bioactive compounds within the context of a balanced diet [10].

Consumer demand for natural products favours the development of bioactive food ingredients with health benefits. Lutein is a promising biologically active component in the food industry, but the new EU regulations regarding the nutrition and health qualifications of foods (EU no. 1924/2006) impose new approaches to support nutritional and healthy qualifications. In this sense, the EFSA Panel on Dietetic Products, Nutrition and Allergies has recently provided a scientific opinion on health claims in relation to lutein and protection of DNA. On the basis of the data presented, the Panel considered that protection of DNA, proteins and lipids from oxidative damage may be a beneficial physiological effect but that a cause and effect relationship has not been established between the consumption of lutein and protection of DNA, proteins and lipids from oxidative damage [11].

In this regard, criteria for supporting health claims of foods have been consensuated and include the characterization of the food component, the inclusion of human intervention trials to probe the effects, and the use of biologically and methodologically validated biomarkers [12], [13]. Within this context, our aim was to preliminary evaluate the safety and potential “functional” effect of a lutein-enriched milk product using the Comet Assay in order to analyze the baseline, the induced DNA-damage and the repair capacity in the lymphocytes of healthy donors before and after the intake of the above-mentioned product. In a former paper, the content, stability, in vitro bioaccesibility and human in vivo response upon the regular consumption of this product has been reported [14].

## Materials and Methods

### Standards and Reagents

Lutein standard (product code X-6250) was purchased from Sigma Aldrich (Madrid, Spain). HPLC grade ethanol, methanol, hexane, methylene chloride and tetrahydrofuran were purchased from VWR Internacional Eurolab (Mollet del Vallés, Spain) and Carlo Erba (Madrid, Spain). Stock solutions of lutein were prepared by dissolving different amounts of the compounds in ethanol, and concentrations were calculated on the basis of absorptivity values (E1% 1 cm; 2550 at 445 nm).

### Lutein Enriched Fermented Milk

As previously reported [14] lutein-fortified fermented milk (Lutein esters mix, Cognis GmbH) was prepared to contain lutein esters at low (equivalent to ca. 4 mg free lutein/100 mL) and high dose (ca.8 mg free lutein/100 mL) according to the protocols and controls required for human consumption. The amounts (ca. 4–8 mg/day) were similar to that contained in 150–200 g of (cooked) spinach and much below the suggested safety levels of intake (20 mg/day) [15].

### Subjects and Bioavailability Study

Details of the study design and methods have been reported elsewhere [14]. Briefly, twenty four apparently healthy volunteers (12 men and 12 women, 18–30 years) were selected by non-probabilistic sampling. All the participants were required **(**inclusion criteria) to have biochemical, haematological and serum levels of vitamin A, E and carotenoids within accepted reference ranges [16]. The use of vitamin and/or herbal supplements, dieting, pregnancy, chronic medication or intercurrent disease or infection that could alter the bioavailability or status of the compounds of interest was used as exclusion criteria. Lutein-fortified fermented milk (Lutein esters mix, Cognis GmbH) (100-ml bottles) was prepared according to the protocols and controls required for human consumption. Transport and storage were maintained at 4°C throughout the study and the lutein content was tested using different protocols of extraction and on different days during the study (i.e. stability) [14].

The study consisted of a multiple-dose bioavailability test using lutein ester-fortified fermented milk, at two levels of fortification (ca. 4 mg and 8 mg/100 mL/day), for 14 days. Subjects were allocated to receive either low-dose (n = 12, six women and six men) or high-dose (n = 12, six women and six men) intervention in a random order by a computer-based table of pseudo-random numbers. Additionally, subjects were provided with a list of lutein/zeaxanthin rich foods to avoid during the study and compliance was tested by personal interview (i.e. counting the servings not consumed, if any). No other dietary restrictions were followed and modifications of dietary and lifestyle habits (i.e., dieting, smoking habit, alcohol, antibiotic consumption), 103 and the presence of fever and infections were also monitored through personal interviews and by a Food Frequency Questionnaire [14].Although no side effects were expected from the intervention, subjects were additionally tested for general indexes in routine clinical practice including lipid profile, hepatic and renal function, vitamins A and E, and haematological indexes.

The study protocol was approved by the Comité Ético de Investigación Clínica of the Hospital Universitario Puerta de Hierro. All subjects were informed and gave their signed consent before their inclusion in the study.

### Blood Sample Analysis

Fasting blood samples for biochemical, hematological and lutein analysis were drawn at baseline (day 0) and day 14. A 10 cc blood sample was preserved to isolate lymphocytes for the Comet Assay. Analyses of lutein were performed in serum samples as described by Olmedilla et al [16]. Samples from each individual (obtained before and after the intervention) were analyzed on the same day to reduce analytical variability. The short- and long-term precision and accuracy of the analytical method are periodically verified through our participation in the Fat-Soluble Quality Assurance Program conducted by the National Institute of Standards and Technology (NIST; Gaithersburg, MD). Biochemical and haematological parameters (inclusion, baseline and at the end of interventions) were monitored by the General Biochemistry and Haematology Laboratories of the hospital according to quality-controlled standardised methods.

### Serum Response Selection

Safety and the effect against oxidative stress were studied using the Comet Assay as a biomarker of DNA damage status. Using the serum levels of lutein as biomarkers of exposure (final concentrations reached upon intervention and within subject net increment of lutein in serum), we randomly selected coded blood samples from volunteers (n = 10) who reached serum concentrations of lutein with epidemiological and biological significance; 1) at 0.40–0.45 µmol/l (23–26 µg/dl) (95^th^ of our reference population [16] and 2) serum lutein concentrations between 0.6–1.05 (34–60 µg/dl) µmol/l which seems to be a safe and desirable target with beneficial impact on visual function and, possibly, on the development of other chronic diseases [5], [8].

### Lymphocyte Separation

Blood samples drawn by phlebotomists were collected in 1 purple top EDTA tube from each participant. These were light-protected, and immediately transported at room temperature to be processed for lymphocytes isolation. Lymphocytes were isolated by standard procedures (Lymphoprep™, ATOM, AXIS-SHIELD PoC AS, Norway), pooled at a concentration of 1×10^6^/ml and stored in liquid nitrogen. Immediately before the Comet Assay, frozen lymphocytes aliquots were thaw up at 37°C and washed in PBS. Viability of lymphocytes and cell number were assessed by a cell counter apparatus (Digital Bio, Seoul, Korea). Samples with a viability bellow 85% were excluded of the functional study.

### Alkaline Single-cell Gel Electrophoresis (Comet Assay)

We followed Singh et al. protocol [17] with minor modifications. Super-frosted and pre-cleaned microscope slides (Fisher Scientific, PA) were immersed in 1% normal-melting-point agarose dissolved in phosphate-buffered saline (PBS, pH 7.4) and allowed to dry. Five µL of a lymphocyte suspension containing approximately 20,000 cells were mixed with 0.5% low-melting-point agarose in PBS, kept at 37°C and spread on an agarose pre-coated slide. Agarose was solidified and a third layer of low-melting-point agarose was added. The cells were lysed for 1 h at 4°C in lysis buffer (pH 10) containing 2.5 M NaCl, 100 mM EDTA, 100 mM Trizma base, 10% dimethyl sulfoxide and 1% Triton X-100, rinsed in 0.4 M Tris-HCl (pH 7.5) to remove detergents and salts and placed on a electrophoretic unit (Owl Separation Systems Inc., Portsmouth, NH, USA) without power for 30 minutes in alkaline buffer (300 mM NaOH and 1 mM EDTA, pH>13) at 4°C. Electrophoresis was carried out for 45 minutes at 25 volts, 295–300 mAmp and a recirculating flow of 100 mL/min. All steps were carried out in the dark. Finally, the slides were rinsed in neutralization buffer (0.4 M Tris-HCl, pH 7.5), fixed in cold 100% ethanol and air-dried.

### Induced-damage and Repair Kinetic Experiments

H_2_O_2_ was used as the source of genomic single-strand breaks. After being embedded in agarose on slides, the cells were incubated with H_2_O_2_ on ice at a concentration of 40 µM for 5 min. Untreated controls were prepared in parallel. H_2_O_2_ was washed off with PBS. Rejoining of single breaks was followed by placing the slides in lymphocytes growth media (RPMI) at 37°C in 5% CO_2_ for 5 and 20 minutes before the lysis treatment. The H_2_O_2_ induced-damage was calculated as followed: ID = (DNA damage at time t0 after exposure - DNA damage before exposure)/DNA damage before exposure. For the analysis of repair kinetics, the percentage of residual DNA damage (%RD) at time *t* after H_2_O_2_ induced-damage was calculated as follows: % RD = 100 X [(DNA damage at time t1 after exposure – DNA damage before exposure)/(DNA damage at time t0 after exposure- DNA damage before exposure) [18], where DNA damage at time t0 corresponds to the damage found immediately after treating the cells with H_2_O_2_ and DNA damage at time t1 corresponds to the DNA damage found after allowing the cells to recover from the H_2_O_2_ induced damage in the lymphocytes growth media.

### Scoring of DNA Damage

Slides were stained with 50 µl of a 1 µg/mL ethidium bromide solution for 5 minutes. Observations were made at 20× magnification using a fluorescent microscope (Olympus BX51 fluorescent microscope, Olympus España, S.A.), connected to a CoHu 4912 CCD camera (CoHu, Inc. San Diego, CA). One hundred consecutive cells (50 from each duplicate slide) were randomly selected avoiding borders and quantified by Komet 5.5 image analysis software (Kinetic Imaging Ltd., Nottingham, UK). Non-detectable cell nuclei or so-called ghost cells, clouds or hedgehogs were not scored, but recorded independently as a percent from the total cells in the slide. The extent of damage was measured quantitatively by the tail moment -TM- (the product of the percentage of DNA in the comet tail and the tail length), and the tail intensity -TI- (percentage of DNA in the tail). Results were expressed as the mean and the standard deviation of TMs and TIs of 100 cells from two duplicate slides.

### Statistical Analysis

All data are presented as mean and standard deviations. Differences in levels of lutein in serum, net increments of concentrations and several parameters of DNA damage in lymphocytes were assessed by paired t test. Correlations between lutein concentrations and DNA damage were assessed using Pearson coefficients. Statistical significance was set at p<0.05 and the analysis were performed with SPSS 14.0 statistical software for Windows (SPSS Inc., Chicago. IL, USA).

## Results

**Figure 1:**
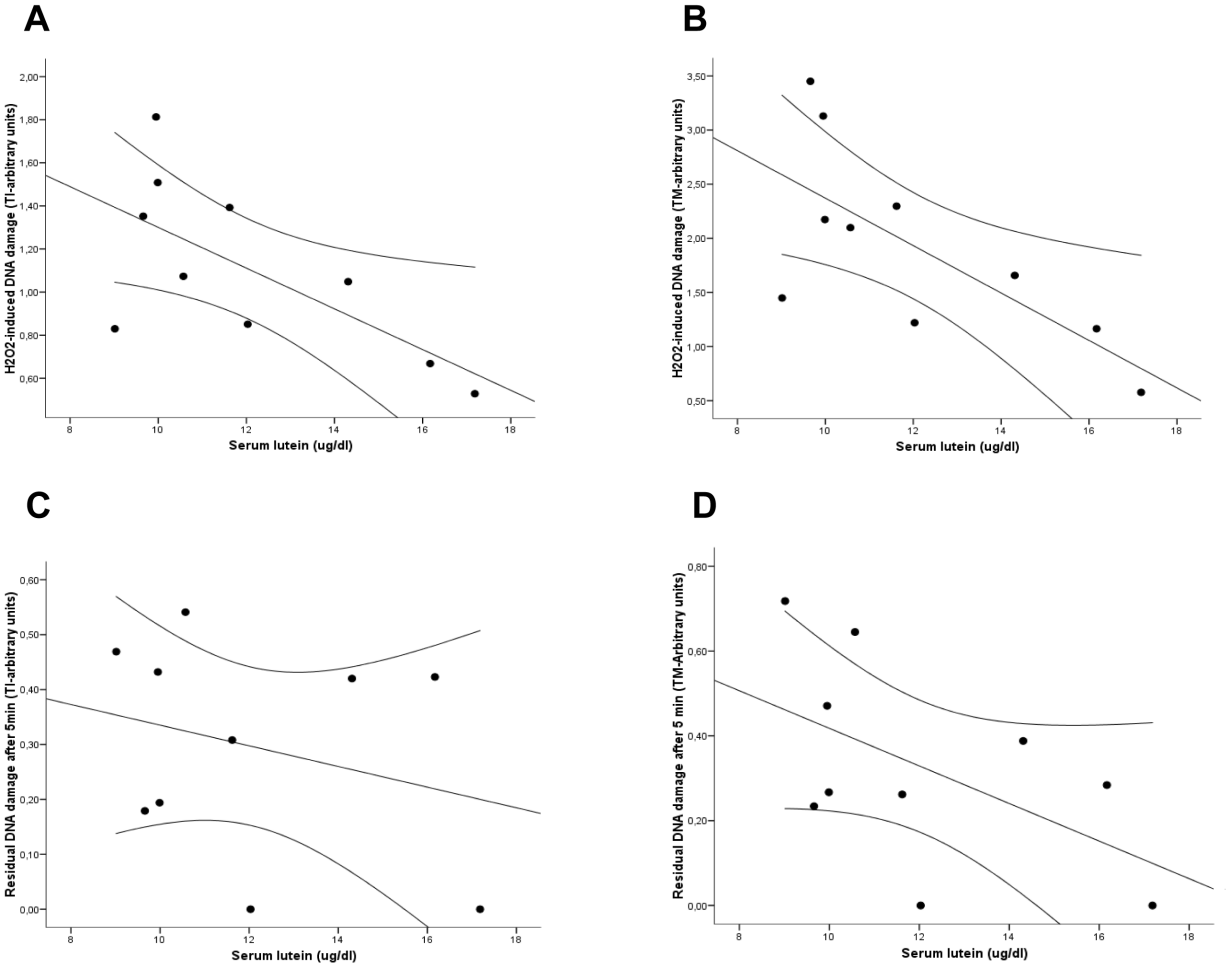
Correlation between baseline levels of lutein in serum and hydrogen peroxide-induced (A, TI value; B, TM value) and residual DNA damage (C, TI value; D, TM value). Lines represent mean fit lines and 95% regression prediction lines.

Baseline serum levels of lutein (before supplementation) did not correlate with endogenous DNA damage (baseline damage) but showed a significant inverse correlation with H_2_O_2_-induced DNA damage in lymphocytes, for both TI (r = −0.67, p = 0.034) (Figure 1A) and TM (r = −0.70, p = 0.02) (Figure 1B). Similarly, the residual DNA damage after 5′of repair was also inversely correlated with basal lutein levels in serum for TI (r = −0.47, p = 0.17) (Figure 1C) and TM (r = −0.65, p = 0.04) (Figure 1D).

After 14 days of the lutein-enriched fermented milk product consumption, serum levels of lutein increased significantly, with a mean net increment above 25 µg/dl and, on average, a 2.5-fold increase after correction for baseline levels (Table 1). Compared to pre-supplementation conditions, endogenous DNA damage and H_2_O_2_-induced DNA damage were lower although differences did not reach statistical significance (Table 1). Lutein levels and net increments achieved in serum were inversely associated with basal DNA damage for TI, although the correlation was not statistically significant (r = −0.33, p = 0.35 and r = −0.32, p = 0.37, respectively). No correlations were observed for the H_2_O_2_-induced DNA damage and lutein levels achieved-serum or with the net increments.

**Table 1:** Serum levels of lutein and DNA damage in lymphocytes of volunteers upon consumption of lutein-enriched fermented milk.

	Baseline (day 0) (mean ± SD)	After lutein-enriched food consumption (day 14) (mean ± SD)
Serum lutein (ug/dl)	12. 1±2.9	37.9±13.8*
Net increment of lutein (ug/dl) (day 14- baseline)	–	27.4±12.8
Net increment/baseline level	–	2.5±1.5
Endogenous DNA damage	21.8±6.1	19.2±3.7
Hydrogen peroxide-induced DNA damage	45.2±12.9	39.2±6.3
Increment of DNA damage	23.4±9.1	19.9±6.4
DNA damage after 5′ repair	27.8±4.0	28.7±2.8
DNA damage after 20′repair	28.4±7.4	29.5±4.7

Values represent “tail DNA” (%) (n = 10).

* p<0.001 (Paired T test).

**Figure 2:**
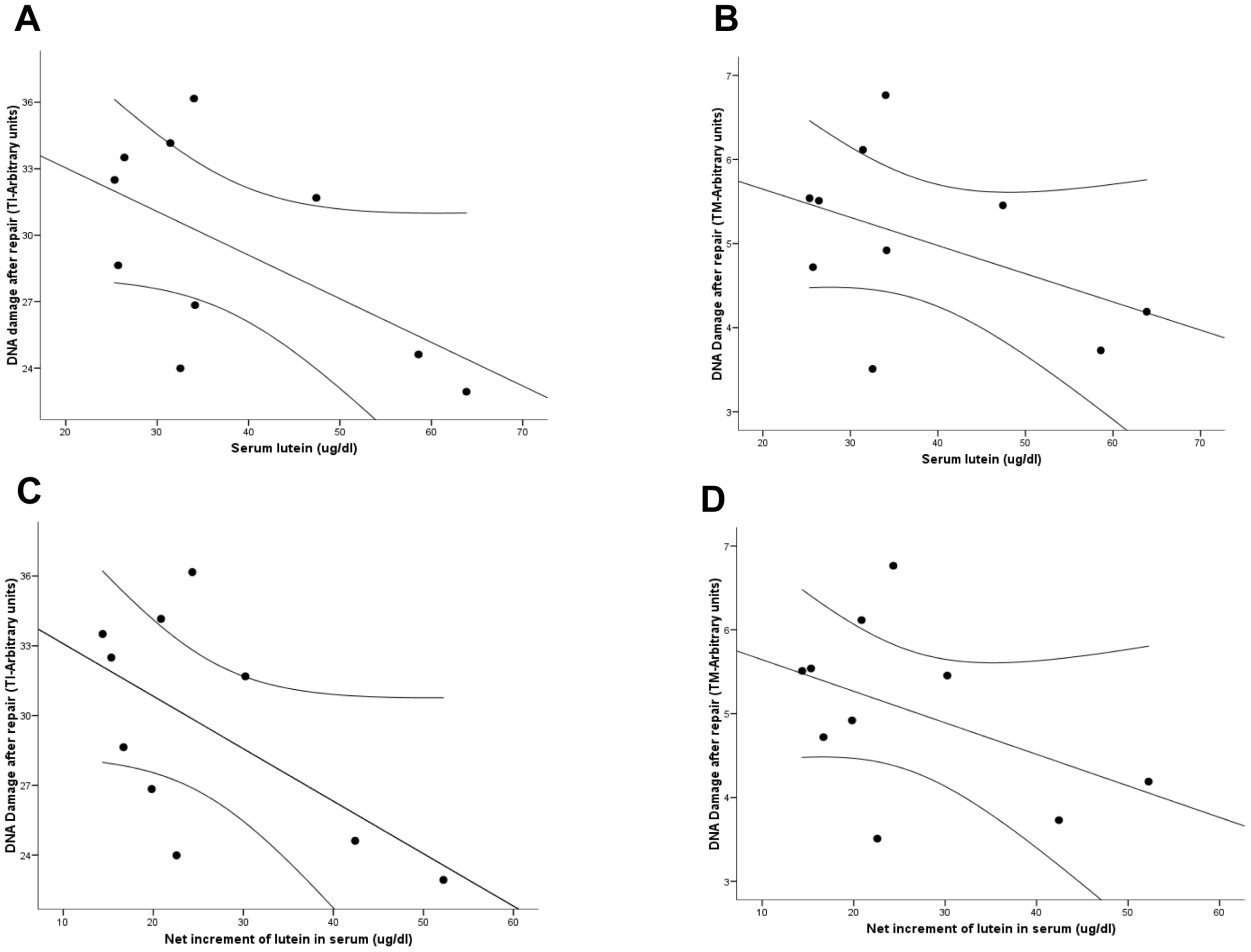
Relationships between serum levels (A, TI value; B, TM value) and net increments (C, TI value; D, TM value) of lutein after intervention and DNA damage after 20′ of repair. Lines represent mean fit lines and 95% regression prediction lines.

Residual DNA damage after 5 and 20 minutes of repair did not change after the intervention (Table 1). However, an inverse relationship was found between the final levels and net increments achieved and the residual damage after 20′ of repair (Figure 2). The correlation coefficient with the lutein levels achieved was r = −0.58, p = 0.08 and r = −0.45, p = 0.19, for the TI and the TM respectively (Figure 2A, B). For the lutein net increments, the correlation was r = −0.59, p = 0.07 and r = −0.45, p = 0.19, for the TI and TM, respectively (Figure 2C, D). Moreover, a trend in the TI parameter between the number of times that the basal levels were increased and the DNA damage after 20′of repair was also observed (r = −0.52, p = 0.12), suggesting a relationship between the magnitude of change in lutein serum levels and the efficiency of the DNA repair.

Within-subject, levels of lutein in serum, endogenous DNA damage and DNA damage after 5′ of repair also correlated before and after supplementation (r = 0.58, p = 0.08; r = 0.53, p = 0.16 and r = 0.68, p = 0.03, respectively), suggesting a strong individual component in the response upon supplementation and the capacity for DNA repair.

Finally, no changes in the biochemical or haematological profile of the subjects were observed during the study, no significant changes in the serum status of vitamin A (retinol) or E (α-tocopherol) were found either and no hypercarotenemia or carotenodermia was observed or reported.

## Discussion

Nowadays, the food industry is increasingly developing and marketing new products containing biologically active compounds with the aim of promoting health and preventing disease. For this purpose, however, *in vivo* actions need to be proven in human subjects under physiological conditions in order to support nutritional and healthy qualifications of foods [11]–[13]. Within this context, in the present study, we have used an experimental food product (lutein-enriched fermented milk) to assess the *in vivo* biological effect of lutein in humans using biomarkers of exposure and of target function. Serum levels of lutein are considered very helpful in assessing compliance and efficacy of intervention and, although they show a wide variability, they are extensively used as the “best available” method to establish exposure and assess the nutritional status [5], [19], [20]. Additionally, the Comet Assay is considered a useful technique to evaluate the amount of DNA damage in practically all cell types and to monitor the effect of dietary compounds on DNA damage and repair capacity [3], [21].

In our volunteers, high lutein levels at baseline (before supplementation) associated with a better resistance to the H_2_O_2_-induced DNA damage, as previously reported [22]. In addition, we observed an inverse correlation between lutein basal levels and the residual damage after 5 minutes of repair. These findings suggest that lutein could neutralize the genotoxic effect of the H_2_O_2_, probably through its antioxidant function, and modulate the cellular DNA repair capability. The fact that both, basal and H_2_O_2_-induced DNA damage associated inversely with lutein levels after supplementation also support this idea. In addition, our results support the lack of a genotoxic effect at the doses assayed and the lutein levels achieved. Although the repair capability of the DNA induced-damage did not change after the intervention, there was an inverse relationship between the residual damage after the repair process and the serum levels achieved, the net increments obtained and the number of the times the baseline levels were increased. These findings are consistent with observations from Zhao et al. [23] who suggested that high endogenous damage due to low fruits and vegetables intake allowed a significant improvement in endogenous repair as soon as 15 days upon supplementation with mixed and single carotenoids. Notably, in the present study, these effects were observed at serum concentrations of lutein similar to those reported by Zhao et al [23], levels consistent with those having a beneficial effect in epidemiological studies [5] but, most importantly, using a food-based approach and at doses compatible with a balanced diet.

Although the present results concern to a small number of subjects and may explain why some changes did not reach statistical significance, the data obtained are consistent with *in vitro* and intervention studies that show an antioxidant and beneficial effect of carotenoids on the DNA repair capacity [24]–[26] although conflicting results have been also found. An inverse association between carotenoids and DNA damage in a representative group of healthy adults have been reported [27]. Similarly, the frequency of chromosome translocations, a biomarker of cumulative DNA damage, was significantly and inversely associated with the intakes of lutein-zeaxanthin [28]. In healthy males, supplementation with spinach products (a dietary source of lutein) resulted in significantly decreased levels of endogenous strand breaks [24]. By contrast, different levels of vegetables and fruits consumption and the corresponding plasma carotenoid concentrations achieved, did not result in differences in the levels of endogenous DNA strand breaks and oxidative DNA damage among healthy, well-nourished, non-smoking men [29].

It is noteworthy that the beneficial effect on DNA damage was observed at serum levels of lutein (marker of exposure) achieved by a food-based approach and, in this sense, our results are consistent with previous data which suggest that a higher benefit may be found in subjects with higher endogenous damage due to lower fruit and vegetable intake [23] while, for people with adequate intake, there is no additional protective effect from supplements beyond that provided from foods [22], [28]. In this context, it should be highlighted that the present study suggests a beneficial effect associated with the consumption of lutein-enriched fermented milk within a balanced diet but any therapeutic use, even at the doses assayed, can not be supported by the present results.

In conclusion, our results suggest that the regular consumption of lutein-enriched fermented milk results in a significant increase in serum lutein levels and this change is associated with an improvement in the resistance of DNA to endogenous damage and the capacity of DNA repair in lymphocytes. However, while the use of a food-based approach in the present study does not exclude completely the possibility of an effect from other food components (i.e. collinearity of nutrients ingested), the dietary control during the study (i,e exclusion of other relevant lutein sources) and the use of serum levels of lutein as a marker of exposure suggest a direct relationship between a change in exposition (lutein intake) and the effect in a target function (protection against DNA damage and improvement in the repair capacity), at least within the range of serum concentrations achieved. Finally, our data also support the lack of a genotoxic effect at the doses supplied as well as the absence of interactions and side effects on other nutritional and biochemicals markers.
